# Bioengineering to Accelerate Biodiesel Production for a Sustainable Biorefinery

**DOI:** 10.3390/bioengineering9110618

**Published:** 2022-10-27

**Authors:** Dheeraj Rathore, Surajbhan Sevda, Shiv Prasad, Veluswamy Venkatramanan, Anuj Kumar Chandel, Rupam Kataki, Sudipa Bhadra, Veeranna Channashettar, Neelam Bora, Anoop Singh

**Affiliations:** 1 School of Environment and Sustainable Development, Central University of Gujarat, Gandhinagar 382030, Gujarat, India; 2 Environmental Bioprocess Laboratory, Department of Biotechnology, National Institute of Technology, Warangal 506004, Telangana, India; 3 Division of Environment Science, ICAR—Indian Agricultural Research Institute, New Delhi 110012, Delhi, India; 4 School of Interdisciplinary and Transdisciplinary Studies, Indira Gandhi National Open University, New Delhi 110068, Delhi, India; 5 Department of Biotechnology, Engineering School of Lorena (EEL), University of São Paulo (USP), Estrada Municipal do Campinho, Lorena 12602-810, SP, Brazil; 6 Department of Energy, Tezpur University, Napaam, Tezpur 784028, Assam, India; 7 Environmental and Industrial Biotechnology Division, The Energy and Resources Institute, Lodhi Road, New Delhi 110003, Delhi, India; 8 Department of Scientific and Industrial Research (DSIR), Ministry of Science and Technology, Government of India, Technology Bhawan, New Mehrauli Road, New Delhi 110016, Delhi, India

**Keywords:** biodiesel, bioengineering, biorefinery, waste, valorization, life cycle assessment, sustainability

## Abstract

Biodiesel is an alternative, carbon-neutral fuel compared to fossil-based diesel, which can reduce greenhouse gas (GHGs) emissions. Biodiesel is a product of microorganisms, crop plants, and animal-based oil and has the potential to prosper as a sustainable and renewable energy source and tackle growing energy problems. Biodiesel has a similar composition and combustion properties to fossil diesel and thus can be directly used in internal combustion engines as an energy source at the commercial level. Since biodiesel produced using edible/non-edible crops raises concerns about food vs. fuel, high production cost, monocropping crisis, and unintended environmental effects, such as land utilization patterns, it is essential to explore new approaches, feedstock and technologies to advance the production of biodiesel and maintain its sustainability. Adopting bioengineering methods to produce biodiesel from various sources such as crop plants, yeast, algae, and plant-based waste is one of the recent technologies, which could act as a promising alternative for creating genuinely sustainable, technically feasible, and cost-competitive biodiesel. Advancements in genetic engineering have enhanced lipid production in cellulosic crops and it can be used for biodiesel generation. Bioengineering intervention to produce lipids/fat/oil (TGA) and further their chemical or enzymatic transesterification to accelerate biodiesel production has a great future. Additionally, the valorization of waste and adoption of the biorefinery concept for biodiesel production would make it eco-friendly, cost-effective, energy positive, sustainable and fit for commercialization. A life cycle assessment will not only provide a better understanding of the various approaches for biodiesel production and waste valorization in the biorefinery model to identify the best technique for the production of sustainable biodiesel, but also show a path to draw a new policy for the adoption and commercialization of biodiesel.

## 1. Introduction

**Figure 1:**
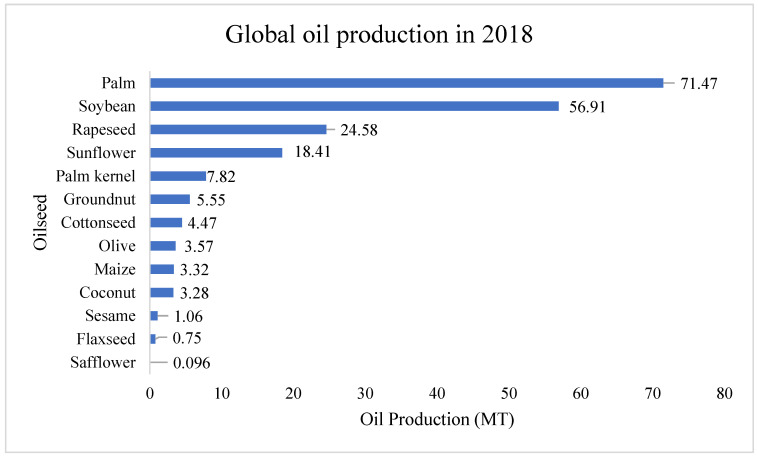
Global oil production in 2018 from oilseed crops. Adapted with permission from Nehmeh et al. [13].

The increasing GHG emissions and depleting fossil-based energy resources require potential and environmentally sound sustainable energy alternatives to overcome these global problems. Surging energy demand owing to rapid population growth, industrial and economic development, accelerated urbanization, and technological advancement requires more energy harvesting from all available sources. Energy consumption was increased from 109,583 terawatt hours in 2000 to 162,194 terawatt hours in 2019 [1] and it is predicted to be 50% higher than the present consumption by 2050 [2]. Simultaneously, the increase in CO_2_ emissions needs to be controlled to prevent climate change. Achieving CO_2_ emission within the range of a ‘safe zone’, i.e., 450 ppm, requires an emission reduction of 50–85% by 2050 [3]. The report of Renewable Energy Policy Network (REN) indicated that around 80% of primary energy comes from the fossil-based resources [4], which is the major cause of GHG emissions. Thus, the emissions of carbon dioxide (CO_2_) from energy-related applications is predicted to continue to increase globally [5]. Biobased energy alternatives are showing potential and gaining significant global attention to replace fossil fuels and resolve concerns regarding climate change. Biodiesel is the most tested and prominent biofuel with similar a composition and combustion properties to fossil diesel. It can be blended or directly used without modifying the engine [6,7]. Various first, second and third-generation feedstocks for biodiesel production have led to promising results [8] and biodiesel produced from various edible and non-edible (energy) crops has already been commercialized [4,9]. Globally, the European Union, USA, Brazil, Argentina, Indonesia and other countries constitute 43%, 15%, 13%, 13%, 6%, and 10% of biodiesel production, respectively [10]. Figure 1 showed the global status of crops used for biodiesel production. However, the debate over food or fuel has been a major concern to overcome for the development of biodiesel [11,12]. Further, land use changes, monocropping for energy crops that caused an impact on land fertility, converting grassland and deforestation are often associated with biodiesel [8]. Thus, it is essential to explore new approaches, including feedstock and those of technology to advance biodiesel production and maintain its sustainability.

Several potential feedstocks including vegetable oil, animal fat, lipid and fatty acids from algae, yeast and other microbes and pathways have been identified to produce biodiesel without affecting the present agricultural system. However, their production potential necessitates bringing it to a commercial level. The high production cost of biodiesel is a major constraint to substituting corresponding fossil diesel [14]. Approximately 75–80% of the total manufacturing cost of biodiesel is attributed to vegetable oil feedstock, which leads to biodiesel production costs reaching almost double that of commercial diesel costs [6,15]. Further, the feedstock with higher free fatty acids, which declines the quality and yield, requires treatment that involves added costs [6]. An economic model study of *Jatropha curcas* biodiesel identified that the expenses would always be greater than income for an annual production capacity of 10,000 m^3^ year^−1^ [16]. Analysis by Sun et al. [17] found significantly higher production costs of microalgae-based biodiesel. This study also identified that the microalgae yield, system operational days are the major obstructions to economical feasible biodiesel production using microalgae. The literature survey demonstrates the need for high lipid production through advancements in technology for cost reduction and popularization of biodiesel.

Genetic engineering methods to produce biodiesel from various sources including plant, yeast, algae, and agricultural or other waste is one of the recent technologies, which could be a promising alternative for creating truly sustainable, technically feasible, and cost-competitive biodiesel [18,19,20]. The higher production of microbial lipids with lower production input may develop a commercial scalable biodiesel production system. Significant progress in both cellular and bioprocess engineering has been achieved in past decades [20]. The metabolic engineering approach includes improved carbon assimilation, limiting fatty acid flux, higher precursors such as acetyl-CoA availability, higher activity of lipid synthesis enzymes, added gene expression towards lipid synthesis, down- regulating the catabolism of fatty acids by inhibiting β-oxidation or lipase hydrolysis and transcription factor engineering [18,21]. Research showed that the metabolically engineered *Yarrowia lipolytica* can accumulate 70–90% lipids of biomass from glucose only [22,23,24]. The *Y. lipolytica* yeast was also successfully engineered to directly use starch [25] or use both C5 and C6 sugars derived from lignocellulosic biomass [26] for oil production. Similarly, gene overexpression was another bioengineered approach adopted for enhanced lipid accumulation in cells. Overexpression of acetyl-CoA carboxylase (ACC) and acyl-CoA synthetases (ACSs), which are responsible to form malonyl-CoA from Acetyl-CoA and thioesterification of fatty acids with coenzyme A to form activated intermediates, respectively, is another strategy to enhance the fatty acid synthesis [21,27]. This approach is successfully demonstrated in several organisms including plants, bacteria, and fungi [28,29,30]. Enhanced lipid synthesis in microalgae was reported by Reik et al. [31] through overexpression of the zinc-finger protein transcription factor.

As identified previously [26,32], the cost of the biofuel should be competitive with corresponding fossil fuels to achieve maximum success. Major production costs come from the raw material, thus, using cheaper feedstock, such as waste cooking oil, inexpensive sugars or sugars obtained from lignocellulosic biomass or animal fat, is a feasible option and can reduce the total cost of bulk chemicals [14]. Focusing on second and third- generation feedstock with the adoption of a biorefinery approach for biodiesel production could be an economically viable option to enhance its sustainability [33]. Moreover, a sustainability assessment for efficient GHGs saving and energy balance is also needed to develop environment-friendly transportation fuel. This article shall henceforth be a review of state-of-the-art research on accelerating biodiesel production using bioengineering approaches and identifying the gap to make biodiesel a sustainable and cost-effective alternative to fossil fuel through a sustainable biorefinery model.

## 2. Scientometric Analysis

Scientometrics is becoming a leading tool for measuring the value of research activities. It has extensive applications in understanding the structure of a discipline, research trends, impact and networks, growth of knowledge and potentiality of cross-disciplinary/cross-boundary work. It also helps in the decision-making for maximum visibility, the introduction of a new policy, tracking emerging trends and finding niche research areas [34].

**Figure 2:**
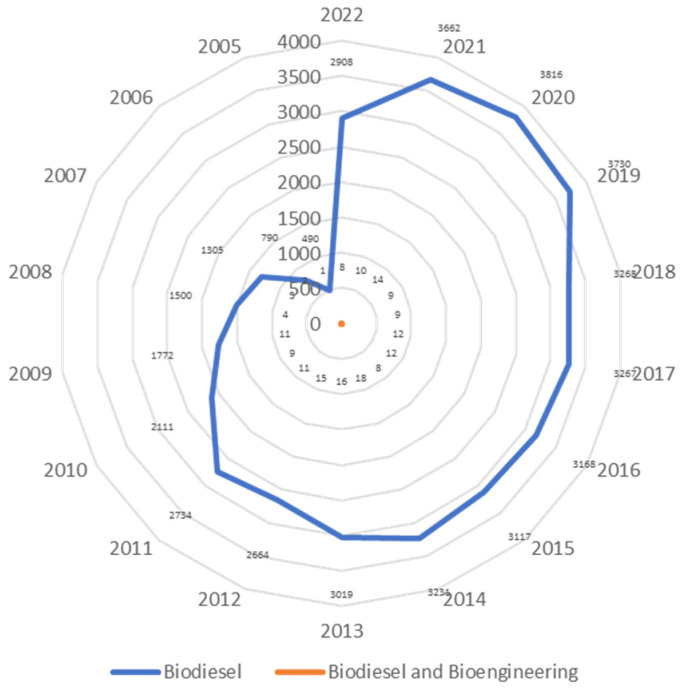
Number of documents published with keywords ‘biodiesel’ and ‘biodiesel and bioengineering’.

**Figure 3:**
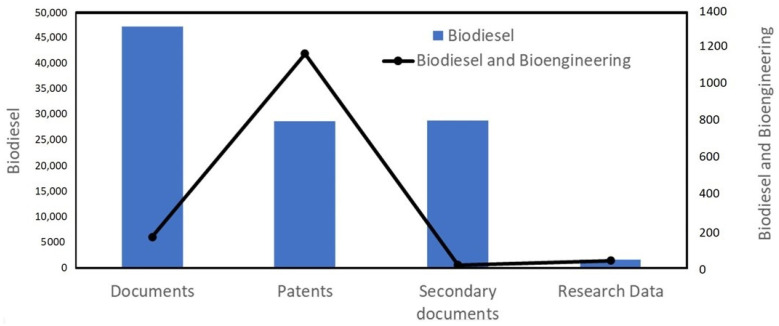
Different types of publications with keywords ‘biodiesel’ and ‘biodiesel and bioengineering’.

**Figure 4:**
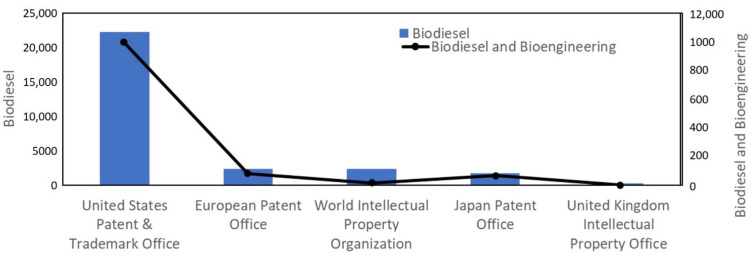
Patents published with different patent offices with keywords ‘biodiesel’ and ‘biodiesel and bioengineering’.

**Figure 5:**
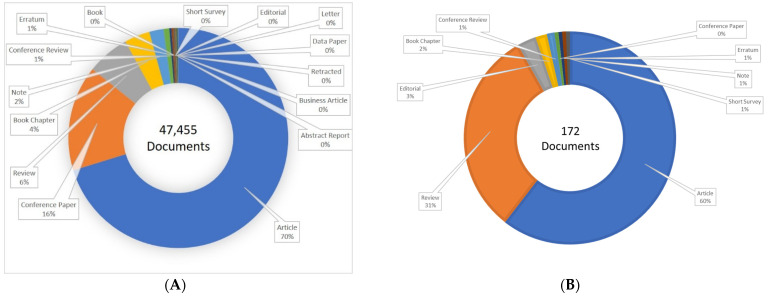
Different types of documents published with keywords (**A**) ‘biodiesel’ and (**B**) ‘biodiesel and bioengineering’.

**Figure 6:**
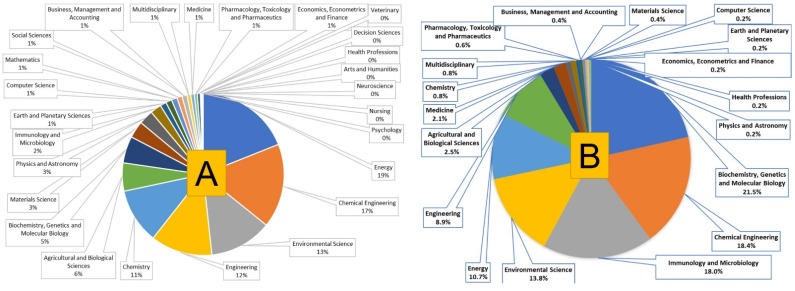
Documents published in different discipline with keywords (**A**) ‘biodiesel’ and (**B**) ‘biodiesel and bioengineering’.

The present scientometric analysis is conducted using the SCOPUS database using the keywords ‘biodiesel’ and ‘biodiesel and bioengineering’. Figure 2 shows that researchers are highly focused on biodiesel research, as about 3000 articles have been published every year since the last decade and researchers have started applying bioengineering tools in biodiesel production. It has been observed that more than 45,000 documents have been published on biodiesel out of which about 200 documents have been published on biodiesel research involving bioengineering (Figure 3). Researchers have published patents for more than 25,000 findings on biodiesel, out of which about 1200 patents covered the bioengineering aspect. The maximum number of patents, more than 20,000 on biodiesel research, out of which about 1000 patents have bioengineering aspect, were published with the United States Patent and Trademark Office (Figure 4). The majority of documents published on biodiesel are articles (70%) followed by conference papers (16%) and reviews (6%), while this trend gets shifted for biodiesel along with bioengineering, maximum documents are article (60%), followed by reviews (31%) (Figure 5). The research on biodiesel along with bioengineering aspects are majorly conducted under energy, chemical engineering, environmental sciences, engineering, chemistry, agricultural and biological sciences, biochemistry, genetics and molecular biology, immunology and microbiology disciplines (Figure 6).

## 3. Approaches to Accelerate Biodiesel Production

**Figure 7:**
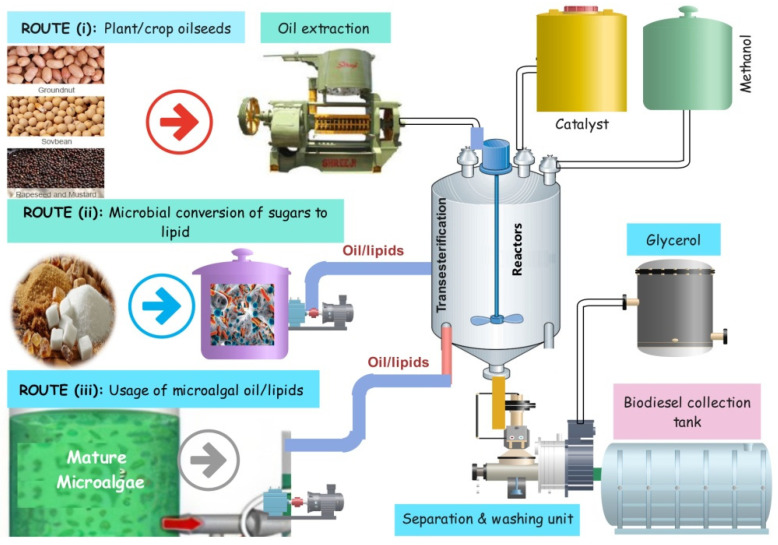
Biodiesel production via the transesterification process through three important routes.

Plants, microalgae, microbes-derived oil, fat, and lipids-based biodiesel are considered the most promising and sustainable feedstock for fossil fuels [25,35]. Biodiesel, as an alternative to fossil diesel, is produced via transesterification mainly by three typical routes [20]: (i) by using vegetable/crop seed oil, (ii) microbial conversion of carbohydrate/sugars to lipid, and (iii) usage of microalgal oil/lipids as shown in Figure 7.

Plant oils, i.e., various edible and non-edible seed oils and many other oil/lipid-bearing materials, are used for biodiesel production [36]. Significant progress has been made so far in biodiesel production from microalgae [37]. However, microalgal oil production using current technologies is still too expensive to commercialize due to efficient photo-bioreactor designs, contamination control methods, and downstream processing. Microalgae biomass cultivation contributes 60–65% of the total production cost for 20–30% of biomass recovery [37,38]. These challenges must be resolved to increase efficiency by incorporating modern tools and bioengineering techniques.

Conversion of sugars/starch/lignocellulose or other C-containing biomass and its bioprocess to lipids through microbial strain bioengineering have several advantages over conventional biodiesel feedstocks. Microbes-based lipids/oils have also been identified as more advantageous in terms of cost-effectiveness, process flexibility, and industrial biotechnology platform for biodiesel production [20,39].

### 3.1. Biomass Selection in a Carbon-Neutral Manner

Biodiesel production with competitive pricing is among the key factor in the bio-based transportation fuels eventually developing the carbon neutral economy. Additionally, biodiesel production contributes to a resource-efficient value-chain in the low carbon fuels and chemicals production. While the dwindling oil prices and political conflicts constituted some constraints in recent years for biofuel production, the bio-diesel industry still seems to have the momentum to continue its production [40,41]. Although a variety of potential feedstock has been evaluated, current biodiesel production processes are not economically and sustainably viable at a large scale, owing to the higher feedstock cost, round-the-year surplus availability of feedstock and energy/cost-intensive processing steps required for biodiesel production. It is evident from some studies that feedstock cost contributes approximately 80% of the total expenditure of biodiesel production [42].

The selection of the right biomass plays a decisive factor in cost-efficient biodiesel production in a carbon-neutral manner. Vegetable oil, non-agricultural (animal fat), Jatropha and biomass-based feedstock are principal resources for biodiesel production in the world. Among the vegetal feedstock, rapeseed, palm, soya, and sunflower are commonly utilized for biodiesel production in the world. Tallow oil (animal fat) and recycled cooking oil are also considered as a sizeable feedstock for the biodiesel production. Rapeseed is the principal feedstock in the world, representing approximately 65% share of global biodiesel production. Palm oil is another major feedstock for the production of biodiesel in countries like Malaysia, Thailand and Indonesia, among others. It constitutes approximately 6% share of biodiesel production in the world. Soybean is the principal feedstock for biodiesel production in the USA and Brazil, constituting around 15% fraction of total biodiesel production [43]. In Brazil, the total production of biodiesel was 5.8 billion liters in 2019, showing an eight percent increase in production relative to 2018. In Brazil, soya alone represents an almost 81.36% share for biodiesel production, followed by animal fat (13.36%), cotton (4.11%), and other fatty material (1.17%) [44]. The use of food resources for biodiesel production developed concerns over food vs. fuel and energy vs. environment. This also caused high food costs and deforestation in turn ameliorating the greenhouse gas emissions [45].

The regular supply of feedstock in large amounts is pivotal to the successful deployment of biodiesel production at a commercial scale. The oil yield in the feedstock (gallons/acre), biodiesel productivity from the feedstock (metric tons/hectare/year), and energy content are the pivotal factors for the selection of an appropriate substrate for biodiesel production. These are trinomial characteristics for commercially successful biodiesel production. The surplus availability of feedstock throughout the year and the cost of feedstocks are the main influencing factors for the successful deployment of biodiesel production in long term. Readily available oil-rich feedstock, for example, soybean or rapeseed looks more viable but in the long term when the concern arises for the selection of food or fuel, then the non-edible feedstock such as *Jatropha curcas*, Karanja or Pongamia oil, Neem oil, Jojoba oil, among others seems more sustainable [46]. Large populous countries such as India and China mainly need to address these feedstocks for biodiesel production. Waste cooking oil is also a substitute feedstock, but impurities and degree of saturation are the possible constraints for using these oils in the transesterification process. The most emerging feedstock for biodiesel production is algal feedstock, which looks more promising in terms of biomass productivity (140–255 Mt/ha/year), high oil content (35–65%), biodiesel productivity (50–100 Mt/ha/year) and high energy content (1150–2000 barrel of oil equivalent/1000 ha/day) [47]. Algal feedstock seems even more financially viable since they are not only high oil-yielding substrates but also high value-added products such as nutraceuticals (biopigments, amino acids, and vitamins, etc.) can also be derived conforming within sustainability guidelines in 3G biorefineries [48]. Lignocellulose feedstocks are also considered important for biodiesel production indirectly. There are plenty of microorganisms which produce oils and lipids and can be grown on lignocellulosic sugars. These microorganisms can be harvested for oil recovery followed by oil transesterification into biodiesel [49].

Considering the current debates on food vs. fuel, energy vs. environment, and the burgeoning demand for transportation fuels, there is a primary concern over the selection of appropriate feedstock for biodiesel production. The use of edible oil as feedstock of biodiesel production may not be a practically viable option. Instead, feedstock such as microalgae, lignocellulosic biomass and used cooking oil are viable options for biodiesel production reducing dependency on conventional diesel eventually contributing to the development of a low-carbon economy.

### 3.2. Constraints

Growing concerns for climate change and increasing energy demand requires the adoption of bioenergy, particularly biofuels. Sustainable alternative energy sources, such as biofuels, limit greenhouse gas emissions and enable a carbon-neutral economy [50]. Nevertheless, the production and utilization of biofuels are influenced by socio-economic environmental and political factors. The primary concern for biofuel production is the choice of feedstock.

Biofuel feedstocks include crops, agricultural by-products, vegetable oils and organic wastes. Based on the types of feedstocks and technology options, the biofuels are grouped into different generations of biofuels. Generally, carbohydrate-rich plant biomass is used as a feedstock for the production of “first-generation biofuels”. However, first-generation biofuels are constrained by socio-economic and environmental issues such as “food security” and greenhouse gas emissions. Lignocellulosic biomass considered second-generation accounts for a major portion (50%) of the total available biomass on Earth [51]. Availability, renewability and cost-effectiveness make the lignocellulosic biomass a valuable feedstock for biofuel production. The biochemical conversion of lignocellulosic biomass involves processes like partial depolymerization of biomass (pre-treatment), formation of simple sugars through the action of enzymes, fermentation of sugars and distillation. Lignin, due to its complex and stable aromatic structures, is recalcitrant to degradation. The pre-treatment methods including the biochemical and physical methods are important to augment the conversion efficiency of biomass [52]. The biological methods are reported to be cost-effective. Huge numbers of microbes including fungi, bacteria and actinobacteria exhibited hemicellulolytic and cellulolytic capabilities [51]. The lignocellulolytic enzymes involved in the biochemical transformation include the hydrolytic enzymes and ligninolytic enzymes [53]. The cost of lignocellulolytic enzymes is a cause of concern for sustainable biofuel production. However, advances in biochemical processes have the potential to increase the conversion efficiency of lignocellulose into simple sugars. Bioengineering of ligninolytic enzymes can augment biofuel production from lignocellulosic biomass [51]. Microbes can be employed for the production of second-generation biofuels by widening the substrate range, increasing their productivity, increasing their tolerance abilities and enabling the production of value-added biochemicals. Metabolic engineering can improve the efficiency of the microbial strain. Metabolic engineering aims to “design native or entirely new metabolic pathways in a cell” [54]. Metabolic engineering improves cellular activities by manipulating the metabolic, transport system and regulatory functions of the cells [50]. Recently, metabolic engineering has been employed to augment biofuel production. Studies have documented the engineering of metabolic pathways for biofuel generation [51,55,56].

The third-generation biofuel feedstock includes cyanobacteria, algae and seaweeds which are a significant source of the production of triglycerides, fatty acids, and lipids. These feedstocks have advantages such as large biomass production and a shorter harvesting cycle [55]. Microalgae, by utilizing sunlight and atmospheric carbon dioxide as energy and carbon sources, respectively, synthesize lipids. The high cost of microalgal production and lipid extraction limits the use of microbially derived lipids for biodiesel production and industrial applications. Challenges in the domain of strain improvement to increase lipid production, growth and substrate requirements, utilization of non-expensive substrates and lipid extraction need to be addressed to augment biodiesel production [55]. The sustainability of algal biofuel production rests on the cultivation system, choice of algal species, source of nutrients, harvesting and downstream processing [57,58]. The production cost of biodiesel using microalgae can be minimized by effective resource utilization, water recycling, and adopting a biorefinery approach [59,60]. To increase the biodiesel production from microalgae, Ranjbar and Malcata [56] suggested genetic engineering measures such as improving lipid yield through manipulation of lipid biosynthesis pathway, increasing metabolic flow towards lipid biosynthesis, lipid secretion, increasing the biomass yield, “transcription factor engineering” and “transporter engineering”.

### 3.3. Bioengineering

Biodiesel contains fatty acid methyl esters (FAME), and it is considered a carbon-neutral fuel as it is made from vegetable oils, hence reducing carbon emission as compared to conventional fossil fuel. Glycerol is widely used in various bioprocess due to its availability and it is also a sub-product of biodiesel production. Khan et al. [61] optimized process parameters for biodiesel production from microalgae using response surface methodology (RSM) and genetic engineering (GA). Carbon dioxide is converted into carbon-rich lipids by microalgae in the presence of sunlight and micronutrients. Microalgae, a non-edible feedstock that can be found in the fresh water, ponds and marine habitats, is advantageous for biodiesel production compared to other high lipid content sources. Process parameter optimization is a very important aspect to produce a high quantity of carbon-rich lipids. The RSM and GA are important techniques to optimize the processes such as molar ratio, reaction time, operating pressure, catalyst concentration, carbon dioxide concentration, pH and temperature.

Singh et al. [62] investigated the thermal pretreatment of bagasse of genetically engineered sorghum for the high recovery of glucose and xylose. Due to advancements in genetic engineering, lipid production is also enhanced in the cellulosic crop and it can be used for bioethanol and biodiesel generation. They also reported that new enzymes can be induced in the cellulosic crops to store more lipids. They used engineered sorghum bagasse as a substrate for lipid and sugar recovery. They used liquid hot water pretreatment for enhancing lipid and sugar recovery and reported that liquid hot water pretreatment at 170 °C for 20 min increases the recovery of lipids and glucose by two-fold [62].

Due to the increase in human population and decrease in cropland and resources, metabolic engineering is required to boost the lipid content in seeds and other plant tissue. In general, more lipid is stored in oil seeds while in other tissue, such as leaves, there is a very minor amount (0.04% to 0.20%) of neutral lipid triacylglycerol (TAG). In the last two decades, metabolic engineering approaches have been studied on various plants to increase the lipid content in plants’ vegetative tissue [63]. In general, three different strategies are used: (i) push: carbon quantity flux is increased through fatty acid and glycolysis pathways; (ii) pull: optimizing TAG assembly; and (iii) protect: reducing the turnover of resulting oil bodies. Vanhercke et al. [64] explained all three different genes that influence stored lipid accumulation in the plant’s vegetative tissues.

Waste cooking oil (WCO) is another alternative for biodiesel generation as it is cheaper and non-edible. The WCO contains a high amount of free fatty acids (FFAs) and its direct use in the transesterification process forms soap in the presence of a base catalyst and reduces the overall efficiency of biodiesel generation. To avoid its soap formation, an acid catalyst can be used to convert FFAs into FAME before the addition of a base catalyst to form TAG. Adding one more step increased the overall cost of the process. To avoid this additional step, lipases can be used to convert both TAGs and FFAs into the FAME in the mild condition of temperature and pH. Heater et al. [65] developed a single step, genetically engineered immobilized lipase for the higher production rate of biodiesel from WCO. They showed that genetic fusion of the *Proteus mirabilis* lipase to Cry3Aa allowed for the production of immobilized lipase crystals (Cry3Aa–PML) directly in bacterial cells. The novel approach showed a 4.3-fold higher enzyme efficiency compared to the conventional lipase enzyme methods. Heater et al. [65] also showed the high activity of Cry3Aa–PML catalyst for 15 cycles for the conversion of WCO to biodiesel. Takeshita et al. [66] genetically muted *Parachlorella kessleri* using heavy-ion beam irradiation for the production of high levels of both starch and lipids. The muted strain is named PK4 and compared to the wild strain it accumulates more lipid, at 1.75 g/L compared to the wild strain of 1.17 g/L. Advanced genetic engineering and metabolic engineering approaches will be used to improve the lipid content in vegetable tissues and can also be used for further biodiesel production. Various researchers reported that the target gene varied with the vegetative part of the plant. Table 1 shows the details of targeted genes responsible for the increase in the lipid content in the plant’s vegetative tissues.

**Figure 8:**
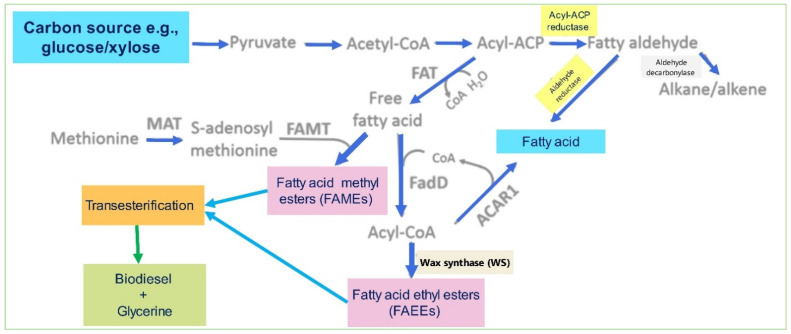
Lipid synthesis pathways to produce biodiesel by *E. coli*.

The production cost of biofuels can be significantly reduced using less biomass expensive materials. However, bacteria or yeasts can convert biomass-derived sugars to lipids/fatty acids that transform into biodiesel via transesterification [20]. Advances in synthetic and metabolic pathway bioengineering have expanded the substrate ranges. *E. coli* was the first microbe studied to produce fatty acid ethyl esters (FAEEs) or methyl esters (FAMEs) directly, which can be used to produce biodiesel. An overview of the synthesis of lipid pathways to produce biodiesel in *E. coli* is shown in Figure 8. The expressions: fatty acyl thioesterases (FATs) lead to the synthesis of free fatty acids (FFAs) converted to fatty acid (FAMEs) by FAMT using AdoMet as FAEEs by acyl-CoA synthase (FadD) and wax synthase (WS). Acyl-ACP is converted to fatty aldehyde by acyl-ACP reductase (ACR) and then to alkanes/alkenes by ADC or fatty alcohols by fatty aldehyde reductase (ALR). Acyl-CoA reductase (ACAR1) can facilitate acyl-CoA conversion to fatty alcohols.

Kalscheuer et al. [72] investigated *E. coli* biosynthesis for FAEEs, and they achieved 1.28 g/L FAEE production in a 2-L fed-batch fermentation. Further, Steen et al. [73] produced biodiesel by engineered *E. coli* and *Z. mobilis*. The engineered *E. coli* was capable of producing FAEEs directly from hemicellulose. Elbahloul and Steinbuechel [74] reported that an engineered *E. coli* can produce FAEEs at a pilot scale with glucose and oleic acid as feeding substances. The classic yeast S. cerevisiae was employed by de Jong et al. [75] to produce FAEEs from ethanol and fatty acyl-CoAs by heterologous expression of a wax ester synthase (WS2) and reported the production of 10 mg/L FAEEs. They suggested examining the direct FAEE production using the oleaginous yeast Yarrowia lipolytica in the future, as S. cerevisiae is not a typical fatty acid producer. Microbial strain engineering, particularly in yeast Y. lipolytica, produces higher fatty acids from various industrial wastes and bio-derived sugars (glucose and xylose) to produce biodiesel. High lipid was produced from xylose by engineered xylose use pathway from lignocellulosic biomass [76].

## 4. Waste Valorization

### 4.1. Current Status

Energy is available in numerous forms, however, population upsurge in conjunction with economic development has imposed a major strain on conventional fuels [77]. To address its repercussion against adverse climatic change, efforts have been directed towards sustainable practices. Sustainable development goals (SDGs) aim to orient economic development with environmental protection owing to the close association of environmental and ecological degradation with extensive economic activities. This has prompted a shift from fossil-based fuels to non-conventional energy sources which includes biomass, solar, geothermal and wind. The energy potential of biomass is considerably high owing to its high availability, lesser emissions along with advanced technologies for its efficient conversion. However, its market share in the energy sector is considerably less [78]. There are a few bottlenecks that exist to obtain the entire energy potential of biomass, thereby, a holistic waste management strategy is needed to recover energy as well as essential nutrients from waste.

Biodiesel is a biodegradable and potential alternative fuel which is derived from renewable sources; however, its large-scale production generates different types of residues such as oil cake and seed kernels in huge quantities after oil extraction. In this context, various research initiatives have been carried out in the waste valorisation of biodiesel residues which offers an excellent opportunity to derive value-added products for different applications.

The various findings regarding the valorization of waste generated during biodiesel production are summarized in Table 2. Oilseed cakes or deoiled cakes and seed kernels are the leftovers generated by extracting oils from them. According to the Food and Agricultural Organisation (FAO)’s Food Outlook November 2020, the worldwide production of oilseed cakes is predicted to be 158.3 million tonnes in 2020–2021 [79]. It includes edible oil cake (e.g., sunflower, mustard, peanuts, soybean) having high protein content along with vitamins and antioxidants that are generally used as supplement feed for cattle, and non-edible oil cake (e.g., castor, neem, mahua, karanja cakes) used in the production of bio-based products such as biofuel, biogas, chemicals, organic fertilizers, pesticides, biopolymer, etc. [80]. Oilseed cakes are an effective way of utilizing agro-waste with an integrated biorefinery approach with the co-production of protein and vitamin-added value products, enzymes, bioethanol, bioplastics and bioelectricity.

De-oiled cake as a biosorbent is studied for decontamination of wastewater or dye in terms of its adsorption behavior. Jatropha oil cake is used as an effective biosorbent in treating aqueous solution containing reactive red dye. The adsorption process was dependent on pH, concentration, temperature, contact time and dose. It was found that at normal conditions (T = 30 ± 2 °C; pH = 7 and 6 h adsorption period), the highest dye adsorption capacity was obtained which was best represented by Redlich-Peterson and Sip isotherms [81]. Hydrolyzed olive cake also exhibited good adsorption-desorption cycles for the removal of copper (II) contaminated fertilizer industry wastewater, with the highest adsorption capacity of 7.32 mg·g^−1^ and the desorption yield changed from 86% to 67.1% [82]. Equilibrium sorption of Cu (II) from synthetic solution by *Jatropha curcas* deoiled cake was higher than Cr (VI) in terms of pH, adsorbent dosage, initial metal concentration and dosage time, which was best fitted by Freundlich isotherm model. Desorption involves the use of chemical reagents such as HNO_3_ [83] or HCl [82] are used for maximum metal recovery. Activation of carbonized oil palm decanter cake (OPDC) exhibited higher adsorption capacities on Cu (II), Pb (II) and Zn (II), but was not found to be suitable for Cd (II) and Cr (VI) adsorption. The adsorption capacities were Pb (II) (128.51 mg/g) > Cu (II) (45.01 mg/g) > Zn (II) (39.21 mg/g), while raw OPDC were more effective in adsorbing Cd (II) and Cr (VI) [84]. Neem oil cake (NOC) finds its wide usage in organic farming as a novel biopesticide and biofertilizer. Additionally, NOC exhibits high adsorptivity for Pb (II) (98%) at low pH (pH = 4) with breakthrough capacity of Pb (II) (30 mg/g) > Cd (II) (15 mg/g) > Cu (II) (10 mg/g) [85]. The occurrence of Ni (II) concentration in the environment from several sources such as metal finishing, tableware plating, forging as well as mine drainage is a serious concern as it may cause several health issues. Jatropha oil cake possesses high affinity to sorb Ni (II) species via different mechanisms, which include ion exchange, chemisorption and physical forces, chelation, complexation and entrapment in the capillaries and pores of the polysaccharide network. Ni (II) adsorption by Jatropha oil cake in its natural form exhibited a removal efficiency of 62% within an hour and 63% in its immobilized form within 90 min [86]. Hydroxyl, carbonyl and carboxyl groups are primarily involved in the biosorption process of Ni (II) [87]. The addition of sodium dodecyl sulfate (SDS) favored the adsorption process of Ni (II) and Zn (II) on carbon derived from mustard oil cake. As revealed by Reichenberg equation, along with pore diffusion, other processes like film diffusion were also the rate-determining steps that were involved during the adsorption process [88]. CO_2_ adsorption was studied using *Pongamia pinnata* seed cake that was processed by hydrothermal and extraction treatments. Breakthrough curves revealed that hydrothermally treated curves exhibited enhanced adsorption capacity, easy desorption as well as good recyclability, thereby proving it to be a promising adsorbent [89].

**Table 2:** Different applications of biodiesel waste through valorization.

Waste from Biodiesel	Type of Feedstock	Applications	Outcomes	Reference
*Jatropha curcas* Deoiled Cake	Non-edible	Adsorption of Cr(VI) and Cu (II) from wastewater	Optimum contact time between adsorbate and adsorbent were 15 min and 60 min for Cr(VI) and Cu(II) respectively Recommended pH of the absorbate were 2 and 6 for Cr(VI) and Cu(II) respectively	Rawat et al. [83]
Oil Palm decanter cake (OPDC)	Edible	Adsorption of heavy metals such as Cu (II), Pb (II) and Zn (II) from waste streams	Maximum adsorption capacities of activated carbon prepared from OPDC were Pb(II) (128 mg/g) > Cu (II) (45.01 mg/g) > Zn (II) (39.21 mg/g) Maximum adsorption capacities of activated OPDC were higher than those of the raw OPDC	Yusoff et al. [84]
Mustard Oil Cake	Edible	Adsorption of Ni (II) from aqueous solution	Optimum pH for biosorption: 8 Highest breakthrough and exhaustive capacities for 10 mg/L Ni (II) concentration were 4.5 and 9.5 mg/g respectively	Khan et al. [87]
Carbon derived from mustard oil cake (CMOC)	Edible	Adsorption of Zn (II) and Ni (II) from aqueous solution	Optimum adsorption capacity of Zn (II) was Ni (II) were 45.8 mg/g and 47.2 mg/g respectively Recovery of Zn (II) and Ni (II) were 75% and 78.97%	Rao et al. [88]
Neem Oil Cake	Edible	Removal and recovery of Cu (II), Cd (II) and Pb (II) from wastewater	Highest adsorptivity was found for Pb (II) (98%) at pH 4 Breakthrough capacities: Pb (II) (30 mg/g) > Cd (II) (10 mg/g) > Cu (II) (10 mg/g)	Rao and Khan [85]
Olive Oil Cake	Edible	Biogas Production	Cumulative yield of biogas: 1226 mL (inoculum ratio: 0.64)	Sarkar [90]
Cotton Oil Cake	Non-edible	Biogas Production	Highest Methane production of 78 mL from 1 g of cotton oil cake	Isci and Demirer [91]
Flaxseed Oil Cake	Edible	Preparation of Spray-dried functional powders for food applications as emulsion stabilizers	Highest stability of the emulsions prepared with the powder was at 200 °C	Drozlowska et al. [92]
Neem Oil Cake	Edible	Evaluation of the effect on plant growth, yield, and management of Alternaria tenuissima leaf spot disease, and rhizosphere microorganisms in chilli crop	Effectiveness in improving plant growth and reducing leaf spot disease: Simarouba > Madhuca > Neem	VasudhaUdupa et al. [93]
Madhuca Oil Cake	Non-edible			
Simarouba	Edible			
Coconut kernel cake	Edible	Used as substrate for lipase production	Highest lipase production: 698 U/g Dry Substrate	Venkatesagowda et al. [94]
Neem Cake	Edible	Used for soil amendment against root knot nematode (*Meloidogyne incognita*) infecting Black gram (*Vigna mungo*)	Effectiveness in plant growth enhancement: Neem > Mustard > Castor > Linseed Neem oil cake was found to be most effective in controlling root knot nematode (*Meloidogyne incognita*)	Rehman et al. [95]
Mustard Cake	Edible			
Castor Cake	Non-edible			
Linseed Cake	Edible			

De-oiled cakes contain micronutrients or different chemicals that might serve as growth enhancers and bio-control agents of beneficial micro-organisms, such as soil bacteria or fungi to antagonize crop-based pathogens and mitigate the use of synthetic agricultural inputs. In a study, four different de-oiled cakes, namely mahua, neem, jatropha and karanja were used as substrate to examine the survival, mass multiplication and population dynamics of *Trichoderma harzianum*. Out of the four substrates, it was found that neem cake was the best substrate in terms of longevity and population dynamics, supporting the survival and growth of *T. harzanium* for more than 105 days, while Jatropha, karanja and mahua cakes could support the growth and longevity of *T. harzanium* for up to 90 days [96]. Eight different oil-seed cakes were used as substrates for lipase production by five different fungi, namely *Chalaropsis thielavioides, Aspergillus niger, Phoma glomerata, Colletotrichum gloeosporioides* and *Lasiodiplodia theobromae.* Out of these, coconut kernel cake as substrate for *Lasiodiplodia theobromae* exhibited maximum lipase productivity, which was further optimized in terms of different operating conditions and the lipase productivity was increased to 698 U/g Dry Substrate [94]. Biopotency of oilcakes, namely neem, mustard, castor, linseed, cotton, olive, flax, soybean, sesame, madhuca and simarouba were also found to increase the yield and growth of *Vigna mungo* [95], tomato [97] and chilli [93] and aided towards direct toxicity and protective action against *Meloidogyne incognita* and *Alternaria tenuissima,* respectively. A reduction in crop productivity and tropical soil fertility causes hindrances to attaining food security. Traditional approaches include the uneconomical usage of mineral fertilizers that cause eutrophication, soil acidity and leaching of phosphates and nitrates that adversely affect the whole ecosystem. Jatropha cake has an abundance of phosphorus, nitrogen, potassium and other organic nutrient sources that improves water, air infiltration and allows for deeper root lengths. Jatropha cake supplemented with compost at different optimized treatment conditions helped to improve the available P, soil pH, exchangeable K, thereby enriching the soil fertility [98]. Nevertheless, Hirota et al. [99] addressed biosafety issues with respect to cake application as soil amendment or biofertilizer. The biopesticidal activity was exhibited by a non-edible oil cake produced from karanja that was used as a substrate for the growth of the fungus *Paecilomyces*. At a C/N ratio of 40:1 and pH = 7, potent termite mortality was observed [100].

Oil seed cakes are used for the preparation of functional powders for use in food industries as emulsion stabilizers. Flaxseed oil cake extract was used as a substrate for the preparation of powder having emulsifying activity. The study investigated the effect of spray-drying process inlet temperature on physicochemical features, regarding oil binding capacity, water holding capacity, solubility, antioxidant activity, chemical composition, surface morphology, color and water activity. It was found that the inlet temperature played a crucial role in the functional and physicochemical properties of the powders in such a way that with the rise in inlet temperature, the antioxidant activity and solubility reduced, but the oil-binding capacity, water-holding capacity, as well as emulsifying activity increased. The highest stability was achieved with emulsions prepared with the powder at 200 °C [92]. Energy production from oil-seed cakes has also been utilized for biogas and hydrogen generation. Biogas production from sunflower oil cakes was in the range of 186–215 mL CH_4_/g volatile solids which reflects its low conversion efficiency that is attributed to the lesser bioavailability of hollocellulose that is interlinked as an intricate polymer of lignin, hemicellulose and cellulose to microbial action. Pre-treatment methods aid to disintegrate these complex polysaccharide linkages to enhance the exposure of fermentable sugars to microbial action. Pre-treatment with 1% H_2_SO_4_ and at 170 °C further enhanced the methane yield to 302 ± 10 mL CH_4_/g volatile solid [101]. Hydrothermal pretreatment enhances cellulose accessibility by increasing the surface area and minimizing the crystallinity of lignocellulosic biomass. Under batch fermentation in mesophilic conditions of sunflower oil cake at 25, 100, 150 and 200 °C, highest methane yield of 310 ± 4 mL CH_4_/g COD_added_ for the liquid fraction and 105 ± 7 mL CH_4_/g COD_added_ for solid fraction was obtained [102]. Hydrogen is an emerging and alternative fuel due to its unique characteristics such as high energy yield and emission of water vapor upon combustion that altogether represents its carbon neutral property. De-oiled jatropha waste was subjected to direct, semicontinuous hydrogen fermentation and the obtained hydrogen yield was 8.7 mL H_2_/g and hydrogen production rate was 1.48 L/L-d when the operational parameters of the reactor were as follows: hydraulic retention time: 2 days, concentration of de-oiled jatropha cake: 200 g/L, pH: 6.5 and temperature: 55 °C [103].

### 4.2. Opportunities

The composition and quantity of oil cake obtained mainly vary with the type of feedstock used, plant growing and processing conditions. Oil cake can be edible or inedible. Edible oil cakes are rich in proteins which adds nutritional value to their use as an animal feed supplement. Oil cakes compose of different nutrients and minerals which makes them a valuable source of nitrogenous fertilizers. Oil cake/meal can be utilized as a substrate for growing microorganisms, and they have been widely used for the production of essential nutrients and chemicals such as amino acids, enzymes, ethanol, organic acids, antibiotics, antioxidants, vitamins, and other bio-chemicals which can be utilized in various foods and pharmaceutical industries [104,105]. Oil cakes and oil seeds are also being investigated as promising sources for the production of biochar, bio-oil and syngas which have many useful applications. Biochar produced can be used as adsorbents for the removal of dyes [106], ion adsorption [107], and many others which highlights the significance of the feedstock. The bio-oil obtained can be upgraded to advanced biofuels and it is a potential storehouse of different chemicals. Oil cakes and oil seeds can be utilized for the generation of hydrogen and biogas and proper integration of biodiesel and non-edible seeds can be used to produce biogas for bioenergy generation which is an economically viable technique [80].

The development of an integrated biodiesel refinery with the simultaneous valorization of its residues and by-products presents opportunities to lower biodiesel production costs as well as the exploitation of biodiesel wastes to derive value-added products is beneficial which increases the overall economic value with reduced emissions and addresses waste disposal issues make biodiesel a sustainable energy resource.

### 4.3. Challenges

As discussed, earlier oil cakes are rich in dietary fibers, proteins and compounds with antioxidant properties, that can be used in bakeries, infant products and supplements [108]. In addition, substrates for producing vitamins, amino acids, antibiotics, enzymes, flavors, pigments, surfactants and bioactive compounds can also be derived from it [109]. Although the production of oil cake is increasing as a result of seed oil production, its application is limited, resulting in low yields from abundant resources. Varieties of applications of the by-products are possible with suitable procedures. Significant pollution is caused by improper management of the oil cakes. The extraction, utilization, and incorporation of dietary fiber and antioxidants in food products need further investigation.

Due to their high nutritional value and moisture content, oil cakes are prone to deterioration. The natural drying is ineffective owing to unpredictable weather and time requirements, causing the product to rot before drying [110]. Hence, effective drying methods with respect to energy, time, cost, and acceptable product quality need to be addressed. Oil cakes also include anti-nutrients such as tannins, phytic acid, antivitamins, saponins and trypsin inhibitors [111]. Although multiple techniques are available for anti-nutrient removal, a common and cost-effective method is yet to be developed.

The high nutritional value and functional benefits of oil cake come with allergenicity due to plant proteins, which stand as a barrier to human consumption. The allergens need to be identified before the introduction of a new protein source as a food ingredient. In addition to that, the digestive aspects of the dose of protein, physicochemical properties and immune response need to be studied in detail. Another challenge with the novel food product is acceptance by consumers. Despite higher nutritional value and added health benefits, not all individuals are willing to try new products, which is known as food neophobia [112].

Biogas production from oil cake is another approach towards value generation from waste. Several factors need to be considered to make it sustainable. Land protection laws in different countries hinder the growth of biogas projects. This is due to increasing population density in certain countries. Thus, biogas production from oil press-cakes needs an in-depth study of social acceptance, related policies and techno-economic feasibility. The supply chain of press cake, including collection, storage and transportation must be clearly identified.

Alkaline-hydrolysis of Jatropha press cake results in a nitrogen source for growing fungi and the production of lipase [113]. Glucose and maltodextrin as carbon sources stimulate fungal biomass formation but decreases lipase production due to catabolite repression. The utilization of alternative carbon sources to overcome catabolite repression and maximization of fungal biomass as well as lipase production needs further research. Oil cakes are not given importance with respect to the amount of bioenergy and valuable products that can be generated. The valorization of oil cakes may help to solve the environmental issue related to waste disposal and support the zero-waste concept. Moreover, to popularize the concept of oil cake valorization, multiple awareness and training programs needs to be conducted. The farmers and industries dealing with seed oil production should be encouraged to learn more about waste valorization and resultant benefits.

As oil cake valorization is a relatively new technology, identification of the supply chain is a major challenge. Technical knowledge towards efficient oil cake generation and its economic value determination is lacking. This will reduce the chances of investment by various stakeholders like farmers, oil millers, traders, power plants and the food industry. Well-defined policies and incentives are also required for the optimal development of this sector. Agri-based products are facilitated by the cooperative expansion of associated organizations [80]. The lack of subsidy, legal issues and lack of cooperative culture in many countries is a serious drawback to the oil cake valorization sector.

## 5. Lifecycle Assessment and Sustainability

Biofuels should be environmentally and economically advantageous to become a sustainable alternative to petroleum [114]. The sustainability of biofuel production systems incorporates energy saving and reduction in greenhouse gas (GHG) emissions and cleaner environmental and social acceptability. A life cycle assessment (LCA) is an internationally recognized tool for determining the associated environmental impacts with all the life cycle stages of biofuel production, processing and utilization, making biofuels more sustainable [8]. LCA is a methodology customarily employed to assess the impact of the product on the environment induced by industrial operations and services, from acquisition, manufacturing, and usage of raw material and its maintenance until the final disposal of products or utilization of services [115]. LCA can also suggest alternative sub-processes to make the process sustainable [35]. With the help of the LCA study, Foteinis et al. [116] explained that the transportation of feedstock is the primary cause of environmental burden for used cooking oil biodiesel, while overall, its environmental sustainability is higher with a 40% reduction of GHGs and ecological footprint. Further, gaining value-added by-products (glycerol, potassium sulfate, and other by-produce) has extra monetary and environmental benefits. Correspondingly, Arguelles-Arguelles et al. [115] conducted an LCA study to produce renewable diesel from palm oil, and reported around a 110% decrease in CO_2_ emissions compared to fossil diesel combustions. Nevertheless, this investigation also reported the impact of palm oil-based biodiesel on the environment and its toxicity to humans due to high agrochemical use in palm plantations.

The LCA of biofuel production systems needs a meticulous design to define the study’s goals, scope, functional units, system boundaries, reference system, comprehensive inventory establishment and detailed information on emissions from products and by-products [117,118]. For example, Larson [119] represented four input parameters that cause more significant variation and uncertainties in LCA results of energy production, which include climate-active plant species (species capability to adjust under altered climate change), N_2_O emission assumption, method allocation for co-product, credits, and dynamics of soil organic carbon. Therefore, in the LCA study, the defining of a goal and scope are two basic steps to determine the system boundary of the study.

Biorefinery is an industrial establishment that sustainably transforms biomass waste materials into biofuels and valuable biochemicals. It is much more complicated than a single biofuel generation system. It is a multi-functional system that creates multiple biochemicals similar to traditional biorefineries. However, it needs a different assessment to determine the goal and scope of such a multi-functional system. In a LCA, various allocation techniques, such as physical or economic allocation are employed to separate the environmental burden of a process or product when numerous functions reflect the same process. Therefore, allocation methods varied by system expansion, monetary value, mass (wet or dry), energy and C-content [114], which may influence the results of LCA studies. Karka et al. [120] assessed the effect of various allocation strategies on LCA results of multi-product biofuel refineries. They figured deviation in the results according to the allocation method followed. Many investigators have recommended adopting an expanded system strategy to compare the environment-related burden of biofuels with fossil fuels [121,122]. In the case when an allocation cannot be avoided, inputs and outputs of the system may be partitioned between various products and by-products [123], which may be performed based on the mass, volume, energy, or C-content of the co-products [124]. The selection of the allocation process dramatically affects the results [121,125]. Allocation based on C-content may be preferred in the investigations pertaining to bioenergy generation because such investigations are targeted to reduce emissions by substituting the traditional fuel resource.

Sheehan et al. [126] conducted a study on LCA to assess energy consumption. They reported that the effectiveness of fossil energy resources using biodiesel produced 3.2 units of fuel product energy per unit of fossil fuel use in LCA. In comparison, fossil diesel LCA produced only 0.83 units of fuel product energy per unit of fossil fuel consumed. Biodiesel use also decreases net CO_2_ emissions by approximately 78.5%, corresponding to diesel per unit of work performed by a bus engine. Such measures confirm the renewable nature of biodiesel. Sieira et al. [127] conducted an LCA study and estimated that biodiesel production generates about 174 times less CO_2_ than diesel production. Nguyen et al. [128] conducted ‘a multi-product landscape life-cycle assessment approach for evaluating local climate mitigation potential’. They reported that the GHG mitigation potential was higher than the C-sequestration value of leaving the corn stover in place for biofuel generation. While intensified and realistic solutions are usually considered through the lens of a stark trade-off, the landscape–LCA results suggest synergies can be achieved when these strategies are combined. Co-adoption of corn-stover collection in fields, no-till system and cover cropping enhances SOC levels, fetch farm revenues, and total landscape production compared to business-as-usual (BAU) management. Malik et al. [129] indicated that the integration of multi-product algal biorefineries and wastewater treatment approach is more sustainable for producing green products in a circular bioeconomy paradigm while keeping the water-energy-environment nexus sustainable. They found a novel and sustainable algal biorefinery route that is more efficient in achieving better biomass valorization by adaptation and use of wastewater for biomass production and efficient removal of N, P, COD, and BOD in the wastewater, sedimentation-based harvesting system, production of alfa-amylase and mycoproteins from residual algal biomass along with recovery of most of the solvents utilized in the process, making it a zero-waste approach.

Singh and Olsen [130], in a LCA study of microalgal biodiesel production considering cultivation of *Nannochloropsis* sp. in a flat-panel photobioreactor (FPPBR), compared six different biodiesel production pathways (three different harvesting techniques, i.e., aluminum as flocculent, lime flocculent and centrifugation and two different oil extraction methods, i.e., supercritical CO_2_ (sCO_2_) and press & co-solvent extraction). They reported that harvesting with lime flocculation and press & co-solvent oil extraction scenarios of biodiesel production provides maximum savings on total impacts. They also reported that scenarios considered in this study also offer GHG savings over fossil diesel, but algal biodiesel is not better in terms of impacts on human health, ecosystem quality, and resources. They suggested that it can be improved by expanding the system boundaries to include the utilization of coproducts and by-products. Arguelles-Arguelles et al. [109] studied the environmental impact of renewable diesel production using an attributional life cycle assessment considering five production stages regarding palm cultivation and harvest, oil extraction, refining, diesel production, and its use and concluded that biodiesel significantly reduces GHG emissions (about 110%) compared to fossil ones. Gupta et al. [131] compared centralized and decentralized rapeseed-biorefinery using the life cycle assessment technique and concluded that centralized biorefinery emitted lesser while energy demand is higher than decentralized ones. They suggested that the system can be improved by the use of low nitrogen nutrients during cultivation, use of biodiesel in farm machineries, improved heating techniques, utilization of waste like glycerol, rape straw, rape cake, etc., and better oil extraction techniques.

LCA can be used as a tool to identify the best combination of various processes involved in the biodiesel refinery by comparing various pathways/techniques available. LCA may also indicate possibilities to improve the system by the adoption of alternate processes/techniques and/or by inclusion of waste valorization within the system boundaries to get sustainable biorefinery.

## 6. Conclusions and Future Prospects

In recent decades, serious efforts have been made to develop strategies to enhance renewable resource efficiency to produce sustainable and eco-friendly energy carriers. Top priority has been given to the compatibility of biofuels with internal combustion (IC) engines. Among the diverse renewable energy resources, biodiesel is reportedly more compatible with IC engines and more environment-friendly. Hence, biodiesel has the capability of meeting energy needs and is also helpful in the transition toward replacing conventional fossil-derived diesel fuels [132]. An increased production of global biodiesel of 19%, from 34.3 billion liters in 2018 to 40.9 billion liters in 2019 was reported in the Renewables 2020—Global Status Report [133]. However, its production is restricted by limited and inadequate raw materials, low economic benefits, lengthy life cycles, impact on food commodities prices, utilization of by-products, net energy ratio (NER) and eco-friendliness limit its application and industrialization.

The extent of these challenges demands several approaches and strategies to capture from the environment and employ bioengineering technologies for efficient waste valorization to establish and achieve circular economy goals [134]. Hossain [135] indicated that the food vs. fuel security conflict might arise due to the utilization of arable land resources for fuel raw materials production. As the oilseed crop remains the primary source of edible oil, its limited oil and lipid production capacity and uncertainty to cater sufficient oils to produce biodiesel in a country like India and China. The utilization of microalgal or lignocellulosic biomass for lipid production is an excellent alternative feedstock. However, lipid production cost and quantity are significant factors behind its applicability. Bioengineering intervention to produce lipids/fat/oil (TGA) and further their chemical or enzymatic transesterification to accelerate biodiesel production has a great future [18,20,62,136]. Microalgal oil production from current technologies is still too expensive to commercialize due to efficient photo-bioreactor designs, contamination control methods, and downstream processing. Such limitations and technological challenges must be tackled, leading to the development of new genetic and bioengineering strategies to improve biodiesel production. Recent advancements in process technology and bioengineering intervention provide promising results in terms of production potential and cost-effectiveness. Microbes, i.e., non-conventional yeast, algae, and bacterial strains, have enormous potential to produce oil/fat/lipids under aerobic fermentation conditions from various economical substrates. Metabolic engineering of microbial strains to synthesize higher lipids and fatty acids by efficiently utilizing substrates has a great future and excellent potential to lower operational costs, improve its economics and accelerate biodiesel production [136,137,138].

The biorefinery for biodiesel and other valuable by-products physically symbolizes the circular bioeconomy concept. While biorefineries are not equally economically strong compared to petroleum hydrocarbon refineries, adoption of a biorefinery model similar to a conventional refinery along with by-products utilization provides economical sustainability through additional income to compensate for the production costs and make it more eco-friendly as emission burdens will be allocated among biodiesel and its by-products. Therefore, biodiesel commercialization will require arduous and combined efforts of R&D work for its capacity for scalability [139]. The biorefinery concept of biodiesel production will also help in improving the NER as some energy inputs will also be apportioned to by-products. A comprehensive environmental sustainability analysis of biodiesel production based on lifecycle assessment (LCA) and various multi-dimensional decision-making techniques would help to develop and prioritize for the achievement of future goals and the aim of sustainable biodiesel biorefinery.

The following outcomes could be made from the present literature survey:

➢ The second and third generation feedstocks are latent resources that can overcome the feedstock restriction.
➢ Advancements in bioengineering approaches used in biodiesel production can enhance production capacity and reduce production costs.
➢ Adopting biorefinery approach provides additional benefit for commercial biodiesel production.
➢ The LCA could be employed as the best tool to identify the best combination of various processes involved in the biodiesel refinery by comparing various pathways/techniques available for the sustainable biodiesel production system.

## Figures and Tables

**Table 1:** Details of targeted genes responsible for the increase in the lipid content in the plant’s vegetative tissues.

Species	Tissue	Target Gene (s)	Total Fatty Acid (TFA) and Triacylglycerol (TGA) Level	Reference
*Arabidopsis thaliana*	Leaf	*LEC*2	TAG not quantified	Santos Mendoza et al. [67]
*Nicotiana tabacum*	Leaf	WRI1, DGAT1, L-OLEOSIN	17.7% TFA (DW), 15.8% TAG (DW)	Vanhercke et al. [64]
*A. thaliana*	Leaf	*PDAT*1	2.6% TAG (DW)	Fan et al. [68]
*A. thaliana*	Leaf	*tgd*1	Not quantified	Xu et al. [69]
*Solanum tuberosum*	Tuber	*ACCase*	0.03% TAG (DW)	Klaus et al. [29]
*A. thaliana*	Seedling	*WRI*1, *AGPase* RNAi	5.8-fold increase	Sanjaya et al. [70]
*Nicotiana benthamiana*	Seedling	*MGAT*2	6.2-fold TAG increase	Petrie et al. [71]

## Data Availability

Data sharing is not applicable to this article.
